# Rat Embryonic Mast Cells Originate in the AGM

**DOI:** 10.1371/journal.pone.0057862

**Published:** 2013-03-07

**Authors:** Michel Farchi Guiraldelli, Carolina Nunes França, Devandir Antonio de Souza, Elaine Zayas Marcelino da Silva, Vanina Danuza Toso, Celiane Cardoso Carvalho, Maria Célia Jamur, Constance Oliver

**Affiliations:** Department of Cell and Molecular Biology and Pathogenic Bioagents, Faculdade de Medicina de Ribeirão Preto-University of São Paulo, Ribeirão Preto, Brazil; National Cancer Institute, United States of America

## Abstract

Mast cells originate from pluripotent hematopoietic stem cells. Two mast cell specific antibodies, mAbsAA4 and BGD6, have previously been used to identify and study committed mast cell precursors (MCcps) in the bone marrow of adult mice and rats. However, the embryonic origin of MCcps is still not known. In the present study, we identified MCcps in rat embryos using these previously characterized mast cell specific antibodies. The MCcps were found in the AGM (aorta-gonad-mesonephros) region of rat embryos at E11.5. These cells were BGD6+, CD34^+^, c-kit^+^, CD13^+^, FcεRI^−^, AA4^−^ CD40^−^, and Thy-1^−^. By PCR the cells contained message for the α and β subunits of FcεRI and mast cell specific proteases. *In vitro*, the MCcps differentiated into metachromatic mast cells. With age of gestation the percent of MCcps diminished while the percent of mast cell progenitors increased. An increased knowledge of the biology and embryonic origin of mast cells may contribute to a greater understanding of allergy, asthma, and other mast cell related diseases.

## Introduction

Mast cells are well known for their involvement in inflammation and allergy [1], and have been recently recognized for their regulatory role in innate and adaptive immunity [2]–[4]. Mast cells in adults originate from pluripotent hematopoietic stem cells [5], [6]. Using two mast cell specific antibodies, mAbAA4 and mAbBGD6, committed mast cell precursors (MCcps, AA4−/BGD6+) have been identified in the bone marrow of adult mice and rats [7], [8]. In adults animals the MCcp appears to be restricted to the bone marrow and does not participate in the mast cell repopulation of peripheral sites [7]. It is the mast cell progenitor (MCp) that is recruited for repopulation of peripheral sites [7]. The mAbAA4 recognizes two derivatives of the ganglioside GD1b [9], [10] that are unique to rodent mast cells [7], [8], [11], [12], while mAb BGD6 binds to a 110 kDa protein on the surface of rodent mast cells [7], [8]. Both mAbAA4 and mAbBGD6 bind to granulated mast cells in all stages of maturation. mAbBGD6, a mast cell lineage marker, also binds to the MCcp [7], [8], [13].

Although the origin of mast cells in adult animals is well established, their exact embryonic origin is still unclear. Using limiting dilution assays, the concentration of mast cell precursors in mouse embryos, embryonic liver and yolk sac has been examined at various days of gestation. The concentration of mast cell precursors reached a maximum at day 11 in the yolk sac and at day 15 in the fetal liver. Furthermore, intravenous injection of 15 day fetal liver cells, but not 11 day yolk sac cells could rescue the mast cell population in mice genetically deficient in mast cells. Therefore, it appears that although the fetal liver contains hematopoietic stem cells that have the capacity to give rise to mast cells, the yolk sac does not possess these stem cells [14], [15]. Further studies have confirmed that cells from the yolk sac of mouse embryos can give rise to mast cells [16]. However, the yolk sac contains pluripotent stem cells that can give rise to many different hematopoietic cell types including mast cells [17]. Although contributing greatly to our understanding of the embryonic origin of mast cells, by relying on indirect methods these earlier studies were unable to conclusively identify the MCcp as well as the site in the embryo where it first appears. In the present study, we have been able to identify MCcps in rat embryos using the previously characterized mast cell specific antibodies, mAb AA4 and mAb BGD6. The difference in binding patterns between the two antibodies was used to identify and isolate embryonic MCcps. The MCcps were initially found in the AGM (aorta-gonad-mesonephros) region of rat embryos at 11.5 days of gestation and at 12.5 days of gestation they appeared in the fetal liver. The phenotype and mRNA expression in the isolated cells confirmed that they are MCcps. *In vitro*, these MCcps differentiate into metachromatic mast cells. An increased knowledge of the biology and embryonic origin of mast cells may contribute to a greater understanding of allergy, asthma, and other mast cell related diseases.

## Methods

### Ethics Statement

All experiments were approved by and conducted under the guidelines of the Animal Ethics Committee of the Faculdade de Medicina de Ribeirão Preto, Protocol number 019/2005.

### Animals

One male Wistar rat (250 g) and three female Wistar rats (200 g) (Animal Facility, Faculdade de Medicina de Ribeirão Preto) were placed in a single cage for mating. On the following morning, the female rats were examined for vaginal plugs to determine the first day of gestation. At embryonic day (E)7.5 to E19.5, the dams were sacrificed and the embryos removed. Dams were euthanized by CO_2_ inhalation followed by cervical dislocation. The intact uteri containing the fetuses were immediately removed from the dam and placed in PBS containing 4%BSA. Individual fetuses were removed from the uterus without disruption of the yolk sac and/or amniotic membrane. For experiments in which preservation of the whole embryo morphology was required, embryos were transferred to PBS+4% BSA at 4°C until anesthesia by hypothermia was completely achieved. When morphological preservation was not necessary, decapitation was used as secondary form of euthanasia for embryos 14 days or older. A minimum of 15 embryos obtained from at least 3 different dams was used at each day of gestation. Bone marrow from adult male Wistar rats was used as a control.

### Antibodies

mAbs AA4, BGD6, and BC4 were kindly provided by Reuben Siraganian (NIDCR, NIH, Bethesda, MD). Normal mouse and donkey IgG were purchased from Jackson ImmunoResearch (West Grove, PA). The mAbs AA4, BGD6, BC4 and normal mouse IgG were directly conjugated to FITC using the FluoReporter FITC Protein Labeling Kit (Invitrogen, Molecular Probes, Carlsbad, CA). Mouse mAb anti-rat CD11b/c (clone OX42) was purchased from Abcam (Cambridge, MA) and rat anti-mouse IgE (Clone LO-ME-2) was purchased from Invitrogen, Biosource. Mouse mAb anti-rat Thy-1 (CD90, Clone HIS51) was purchased from BD-PharMingen (San Diego, CA). Rabbit anti CD40 (C-20, an affinity purified polyclonal antibody raised against a peptide mapping at the C-terminus of CD40 of human origin), goat anti-CD13 (C-17, an affinity purified polyclonal antibody raised against a peptide mapping at the C-terminus of CD13 of human origin), mouse mAb anti-rat CD34 (clone ICO115) and rabbit anti c-kit (CD117, C-19, an affinity purified polyclonal antibody raised against a peptide mapping within the C-terminus of c-kit of human origin) were purchased from Santa Cruz Biotechnology, Inc. (Santa Cruz, CA). The following secondary antibodies were purchased from Jackson ImmunoResearch: donkey anti-mouse IgG F(ab)′_2_-FITC, donkey anti-rabbit IgG F(ab)′_2_-FITC and donkey anti-goat IgG F(ab)′_2_-FITC.

### Coupling of Antibodies to Magnetic Beads

mAb AA4 and mAb BGD6 were conjugated to tosylactivated Dynabeads (Dynal, Life Technologies, Camarillo, CA) as previously described [8], [18].

### Microscopy

#### Tissue

The embryos were removed from the uterus, washed in PBS and fixed in 2% formaldehyde (Electron Microscopy Sciences, Fort Washington, PA) in PBS. Embryos were processed with routine histological techniques and embedded in Paraplast (Oxford Labware, St. Louis, MO). 4–5 µm serial sections were cut and stained with toluidine blue (0.1% toluidine blue and 1% acetic acid, pH 2.8) or hematoxilin and eosin. Alternatively, embryos were frozen in Tissue Freezing Medium (Electron Microscopy Sciences) and 10–15 µm serial sections were obtained from the entire embryo, placed on glass slides, rinsed twice in PBS, fixed with 2% formaldehyde (Electron Microscopy Sciences), blocked with PBS+1% BSA (Sigma Aldrich, St. Louis, MO) and incubated with 2.5 µg/ml mAb AA4 or 20 µg/ml mAb BGD6 directly conjugated to FITC for 3 hours at 37°C. Following incubation, the tissue was rinsed and coverslips mounted with Fluoromount G (Electron Microscopy Sciences). Some sections were stained with toluidine blue or hematoxilin and eosin.

#### Cells

Freshly isolated or cultured cells were placed on Cell Tak (BD Biosciences, San Jose, CA)-coated coverslips. The cells were then rinsed in PBS, fixed with 2% formaldehyde (Electron Microscopy Sciences), followed by rinsing in PBS containing 0.1 M glycine. Non-specific binding to Fc receptors was blocked by incubation with donkey IgG (5 µg/ml) diluted in PBS containing 1% BSA. The cells were then incubated with mAb AA4 (2.5 µg/ml) directly conjugated to FITC, mAb BGD6 (10 µg/ml), anti FcεRI α subunit (10 µg/ml), anti-IgE (10 µg/ml), anti c-kit (15 µg/ml), anti CD34 (15 µg/ml), anti CD13 (20 µg/ml), anti Thy-1 (15 µg/ml), and anti CD40 (10 µg/ml), for 1 hour at room temperature. Following incubation, the cells were rinsed thoroughly in PBS and the samples, except those incubated with mAb AA4, were incubated with secondary F(ab′)^2^ antibody conjugated to FITC. The samples were then rinsed and coverslips mounted with Fluoromount-G (Electron Microscopy Sciences).

#### Controls

As controls, samples were incubated with normal mouse IgG conjugated to FITC, unlabeled normal mouse IgG or without primary antibody. Samples incubated with unlabeled normal mouse IgG or without primary antibody were incubated with labeled secondary antibodies. All controls were negative.

### Imaging

The sections and cells were analyzed using a Nikon Eclipse 800 Microscope (Nikon Instruments, Inc., Melville, NY) with 10×/0.25, 20×/0.40 and 40×/0.75 objectives. The microscope was equipped with a Nikon DXM 1200 digital camera and Act1 software. Images were post-processed using Adobe Photoshop CS5 (Adobe Systems, Inc., San Jose, CA).

### Embryo Dissociation

Embryos at E11.5, E13.5 and E15.5 were removed under sterile conditions from the uterus and immediately placed in PBS+4% BSA (Sigma Aldrich), cut into fragments of approximately 1 mm^3^ with a razor blade, and washed in PBS by centrifugation at 27×*g* for 5 min. The pellet was resuspended in 10 volumes of PBS+0.53 mM EDTA (Sigma Aldrich) and mechanically dissociated using a Pasteur pipette. Additionally, embryos at E13.5 and E15.5 were washed with Iscove's Modified Dulbecco's Medium (Invitrogen) and the dissociation completed by incubating the fragments in media containing 2.5 mg/ml of type III collagenase (Worthington Biochemical Corporation, Freehold, New Jersey) for 45 min at 37°C with constant agitation. This process was repeated twice and the cells pelleted by centrifugation (27×*g*), washed twice with Iscove's medium and counted in a hemocytometer.

### Cell Separation

Embryonic mast cells were sequentially immunoisolated according to the method described by Jamur et al [8]. Briefly, the cell suspension from dissociated embryos was washed twice by centrifugation (27×*g*) in Iscove's Medium containing 2% BSA and 5 µg/ml normal mouse IgG (Jackson ImmunoResearch). The cell suspension was then incubated for 5–10 min at 16°C with mAb AA4 coated beads and the mast cells (AA4+) bound to magnetic beads were removed using a magnetic particle concentrator (MPC) (Dynal). This step was repeated once. The AA4+ mast cells were then counted in a hemocytometer. The AA4−/BGD6+ mast cells were then isolated by incubating the AA4− cell suspension with beads conjugated to mAb BGD6. The AA4−/BGD6+ mast cells bound to the beads were isolated using the MPC and counted in a hemocytometer.

### Cell Culture

The isolated AA4−/BGD6+ mast cells were cultured, (10^6^ cells/ml), in Iscove's Medium supplemented with penicillin (100 U/ml), streptomycin (100 µg/ml), amphotericin B (0.25 µg/ml; Invitrogen, Gibco), 0.05 mM β-mercaptoethanol, 10% fetal calf serum, 100 ng/ml recombinant stem cell factor (SCF) (Invitrogen, Biosource) and 20 ng/ml recombinant interleukin-3 (IL3) (Invitrogen, Biosource). A total of 0.5 ml of complete medium was added every 5 days for 21 days.

RBL-2H3 cells, a rat mast cell line, were cultured as previously described [19]. PC-12 cells, a rat pheochromocytoma cell line were cultured as suggested by the American Type Culture Collection (Manassas, VA).

### Cell Counts

For immunostaining, the data are expressed as the X ±SD% of the total cells that stained with the different antibodies in 7 different experiments. In each experiment at least 100 cells per antibody were counted using fluorescence microscopy in combination with DIC microscopy.

### RT-PCR

Immunomagnetically isolated cells were suspended in 50 µl of PBS followed by the addition of 5 volumes (250 µl) of ice cold RNAlater (Qiagen Valencia, CA,) and immediately stored at −80°C.Total RNA was extracted from 1×10^5^ cells (RBL-2H3 cells, PC-12 cells, isolated bone marrow mast cells AA4+/BGD6+, isolated bone marrow MCcp AA4−/BGD6+ and isolated embryonic MCcp AA4−/BGD6+) using the QIAGEN RNeasy Mini Kit and the quality of the isolated RNA was verified on a 1.2% formaldehyde agarose gel. The first strand cDNA synthesis was obtained with the SuperScript Synthesis Kit for RT-PCR (Invitrogen) with 5 µg of total RNA and primed with oligo DT. Primers (Invitrogen) used for PCR were: FcεRIα – forward AGCAACAACATCTCCATTAG, reverse TTGCCTTTTCCAGTCTTC; FcεRIβ - forward TTCCTGGATAGTCCAATTAATG, reverse GCTCGAGATTGTCCTGGCCCCCTTG; RMCP-1 – forward CCACTGAGAGAGGTTACAAG, reverse – CCACGTCCATAAGATACAATAC; RCMP-5 – forward CAACTTCAACTTCATTCCAC, reverse CTTCAGCAGGAACTACAGAC; R-CPA – forward GTGAAAGAGGAAATCGCAGG, reverse - GAGTCCCATGAGACATCGAAG). The RT-PCR reaction was performed with 10 nM specific primers using the Platinum PCR SuperMix High Fidelity (Invitrogen). The samples were initially denatured at 94°C for 10 min. Amplification was done for 35 cycles of 45 s denaturing at 94°C, annealing for 30 s at 55°C, elongation for 2 min at 68°C, followed by a final extension step of 5 min in a thermocycler (GeneAmp PCR System 9700, PE Applied Biosystems). The PCR products were analyzed on 1.5% agarose gels.

## Results

### BGD6+ cells appear at 11.5 days of gestation in the AGM region of rat embryos

**Figure 1:**
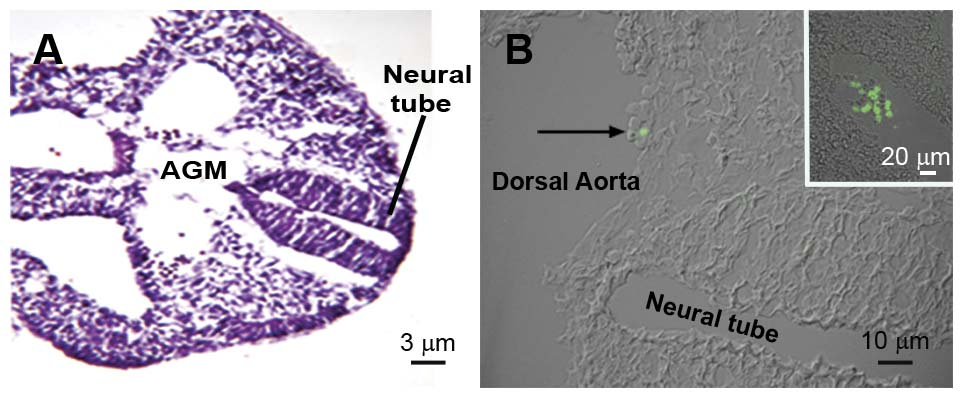
Transversal section of aorta, gonads, and mesonephros region (AGM) of embryos at E11.5. (A) AGM region stained with Hematoxilin & Eosin (H.E) (B) Combined image of Differential Interference Contrast (DIC) and fluorescence microscopy showing a MCcp (arrow) labeled with mAb BGD6-FITC located near the inner face of the dorsal aorta wall. Inset: Clusters of MCcps are occasionally seen in the AGM.

From E7.5 through E10.5 cells immunolabeled with mAbAA4 or mAb BGD6 were only found in the maternal decidua surrounding the embryo and close to blood vessels near Reichert's membrane which separates trophoblast cells on the uterine side and parietal endoderm cells on the embryonic side. In addition, no immunostained cells were observed in the yolk sac at this stage. From E7.5 until E11.5, no immunostaining was observed in the entire embryo with either mAb AA4 or mAb BGD6 (data not shown). At E11.5 cells positive only with mAb BGD6 were identified in the AGM region (Fig. 1). These cells (AA4−/BGD6+) were located near the inner face of the dorsal aorta wall, close to the endothelium. Occasionally, clusters of AA4−/BGD6+ cells were seen in the AGM region (Fig. 1B and inset). At E11.5 no cells immunolabed with mAbAA4 could be identified in these embryos.

### Characterization of AA4−/BGD6+ cells confirms that they are MCcps

**Figure 2:**
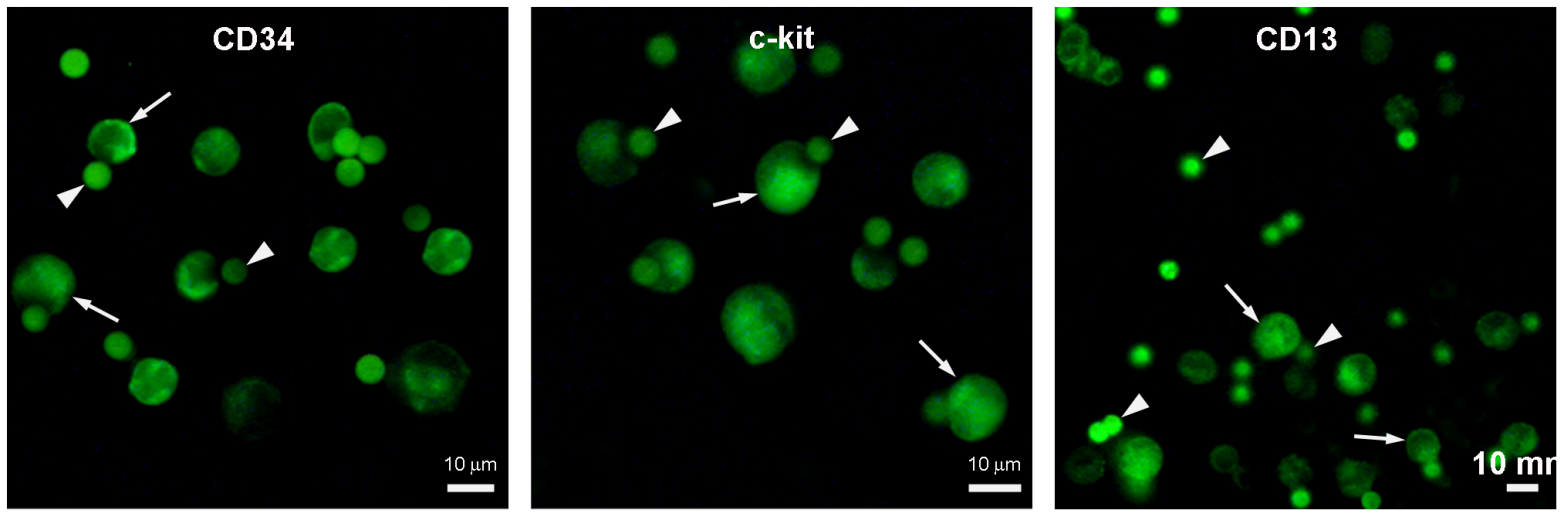
MCcps (AA4−/BGD6+) were immunomagnetically isolated from E11.5 rat embryos. After immunostaining, the cells were positive for the expression of CD34, c-kit and CD13. The secondary antibody was conjugated to FITC. Arrows, immunolabeled cells; arrowheads, magnetic beads conjugated to mAb BDG6.

AA4−/BGD6+ mast cells were immunomagnetically isolated from E11.5 embryos. The AA4−/BGD6+ cells represented 0.19%±0.0027% of the total number of embryonic cells and 99.5±0.3% of the cells stained positively with mAbBGD6. Additionaly, by immunostaining, the cells were 99.1±0.5% CD34^+^, 99.6±0.2% c-kit^+^, 97.8±0.8% CD13^+^ (Fig. 2), CD40^−^, and Thy-1^−^. They did not express the alpha subunit of FcεRI, or the gangliosides recognized by mAb AA4 nor did they bind IgE.

**Figure 3:**
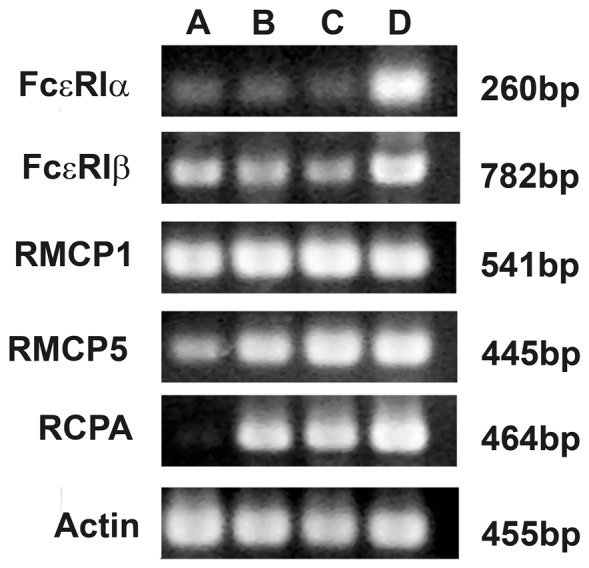
RT-PCR analyses of the expression pattern of the alpha and beta subunits of FcεRI and mast cell specific proteases. Lane A. MCcp (AA4−/BGD6+) isolated at E11.5; Lane B. Adult rat bone marrow MCcp (AA4−/BGD6+); Lane C. Adult rat bone marrow mast cells (AA4+/BGD6+); Lane D. RBL-2H3 mast cells. FcεRIα: α subunit of the high affinity IgE receptor; FcεRIβ: β subunit of the high affinity IgE receptor; RMCP1: rat mast cell protease-1; RMCP5: rat mast cell protease-5; RCPA: rat carboxypeptidase A.

RT-PCR experiments on the freshly immunomagnetically isolated AA4−/BGD6+ mast cells from embryos at E11.5 showed the expression of mRNA for the alpha and beta subunits of FcεRI, rat mast cell protease 1 (RMCP-1) and rat mast cell protease 5 (RMCP-5). However, they did not express significant levels of carboxypeptidase A (CPA) (Fig. 3). Except for the lower levels of CPA expression in MCcps from embryos, a similar pattern of expression was previously seen in immunomagnetically isolated MCcps (AA4−/BGD6+) from adult rat bone marrow, immunomagnetically isolated mast cells (AA4+/BGD6+) from adult rat bone marrow, and in RBL-2H3 mast cells (Table S1). No expression of any of these mRNAs was seen in the PC-12 cells, which were used as a control (data not shown).

**Figure 4:**
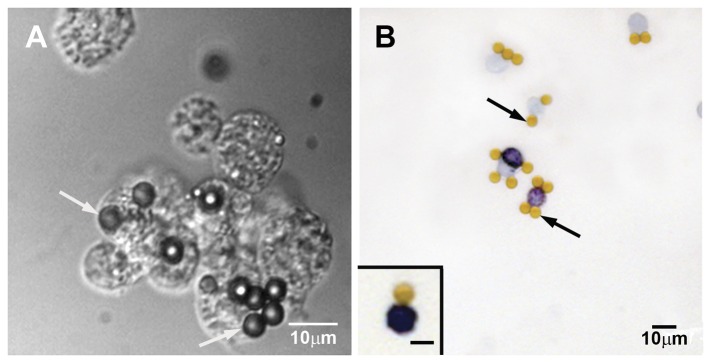
MCcps immunomagnetically isolated with mAb BGD6 from E11.5 embryos. (A) After 21 days in culture with SCF and IL-3 the MCcp grew as small colonies around the immunomagnetic beads (white arrows). (B) After 21 days in culture, many of the mast cells displayed metachromatic granules. Inset: A higher magnification of a metachromatic mast cell attached to a immunomagnetic bead. (Immunomagnetic beads, black arrows).

When immunomagnetically isolated AA4−/BGD6+ mast cells from E11.5 embryos, were cultured with SCF and IL3, the cells grew in suspension as small colonies (Fig. 4A). After 7 days in culture, few metachromatic mast cells could be identified. After 3 weeks in culture, 32.2%±3.5% of the cells displayed metachromatic granules (Fig. 4B). By immunolabeling, all cells expressed the α-subunit of FcεRI, c-kit, and the gangliosides derived from GD1b. Additionally, the cells bound IgE on their surface. Therefore, taken together, these results indicate that the immunomagnetically isolated AA4−/BGD6+ mast cells from E11.5 embryos can be classified as MCcps.

### AA4+ mast cells first appear in the liver at E12.5 and subsequently, colonize other sites in rat embryos

**Figure 5:**
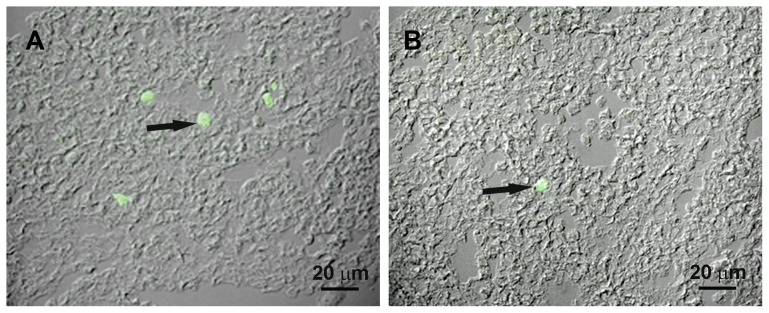
Representative fields of liver from E12.5embryos. (A) At this stage, several mast cells are labeled with mAb BGD6 conjugated to FITC (arrows). (B) In contrast, only one immature mast cell (arrow) is immunolabeled with mAb AA4 conjugated to FITC.

**Figure 6:**
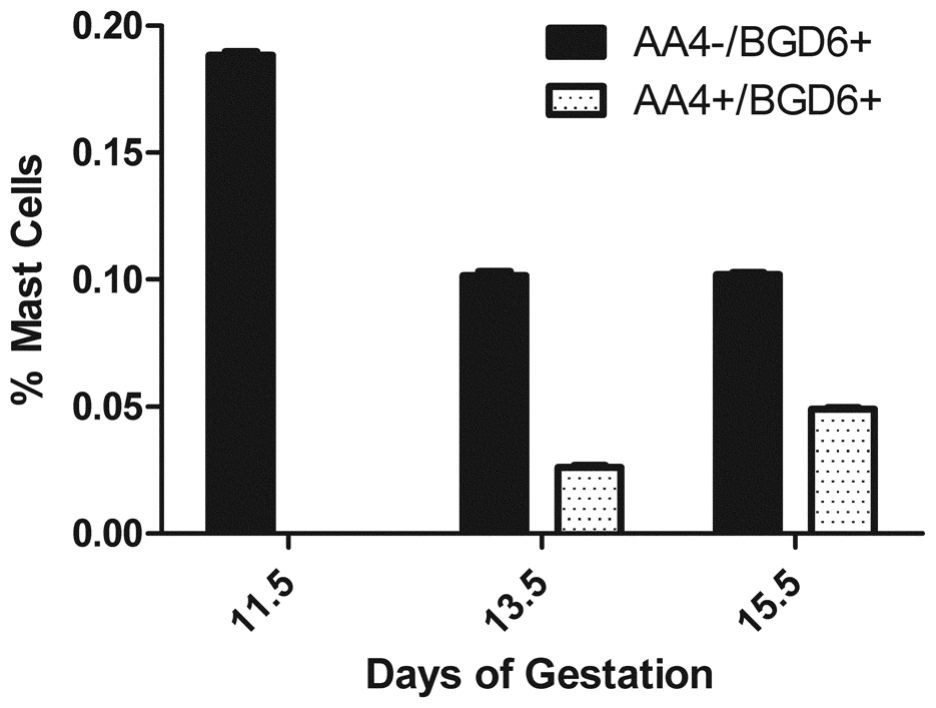
AA4−/BGD6+ and AA4+/BGD6+ cells were immunomagnetically isolated from dissociated embryos. With increasing days of gestation the percent of AA4−/BGD6+ mast cells decreases and the percent of AA4+/BGD6+ mast cells increases. Data is expressed as the mean±SD of 6 independent experiments.

At E12.5, MCcps were present in the liver (Fig. 5A). However, an occasional immature mast cell (AA4+/BGD6+), presumably a mast cell progenitor (MCp) could be identified, but exclusively in the rat embryonic liver (Fig. 5B). The MCps were present in lower numbers than the MCcps. The MCps did not contain metachromatic granules and could be identified as mast cells only by immunolabeling. With increasing days of gestation the percent of MCcps decreased in the embryos, while the percent of AA4+/BGD6+ mast cells increased (Fig. 6).

**Figure 7:**
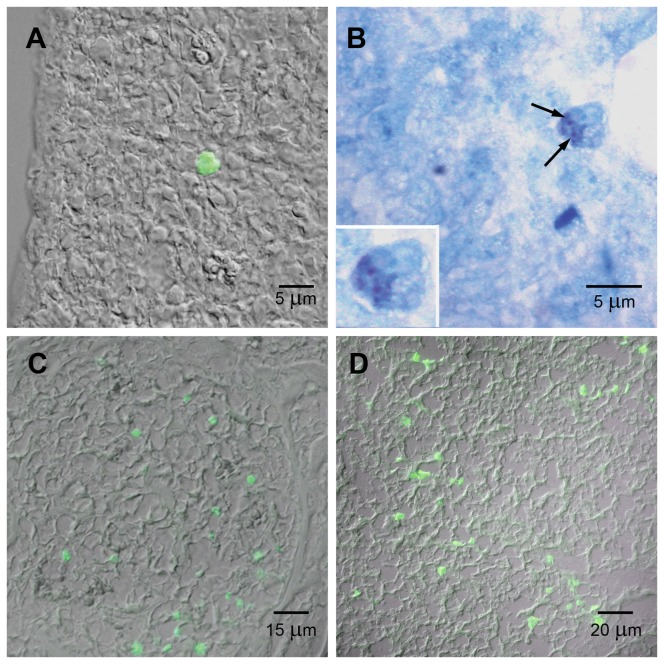
From E13.5–19.5 mast cells colonize peripheral sites. Very immature mast cells (AA4+/BGD6+) could be identified by immunostaining in the caudal mesenchyme (A) at E13.5. At E15.5, the first metachromatic mast cells (arrows) appeared in the caudal mesenchyme (B). At E18.5 mast cells first appear in the bone marrow (C). At this time mast cells could only be identified in the bone marrow and in the liver (D) by immunostaining. A, C and D immunostained with mAb AA4-FITC. B, toluidine blue.

The colonization of additional sites, other than the liver, began at E13.5. The kinetics of colonization of embryonic tissues by very immature mast cells (AA4+/BGD6+) in embryos ranging from the E12.5 to E19.5 is shown in Table 1. At E13.5 when very immature mast cells (AA4+/BGD6+) appeared in the dorsal and caudal mesenchyme, they could only be identified as mast cells by immunostaining (Fig. 7A). At E15.5, the first metachromatic mast cells, also AA4+/BGD6+, appeared in the caudal mesenchyme (Fig. 7B), and in the skin next to the follicles of vibrissae (not shown). By E17.5 metachromatic mast cells could be found in the majority of organs. However, at E18.5 when immature mast cells (AA4+/BGD6+) first appear in the bone marrow (Fig. 7C), metachromatic mast cells cannot yet be found in either the bone marrow (Fig. 7D) or liver.

**Table 1:** Kinetics of colonization of peripheral tissues/organs.

Days of gestation								
11.5	12.5	13.5	14.5	15.5*	16.5	17.5	18.5	19.5
***MCcp*** 1	***Maturing mast cells*** 2							
AGM	Liver	Mesenchyme,	Lung	Intestine	Adrenal	Pancreas	Bone marrow	Connective tissue^3^
		Mesencephalon	Skin		Kidney	Salivary glands	Thyroid gland	
			Digestive tube		Thymus	Tongue		
			Follicles of vibrissae					

* First appearance of metachromatic mast cells.

1 *AA4−/BGD6+.*

2 *(AA4+/BGD6+).*

3 near blood vessels.

## Discussion

In this study, for the first time, the site of origin of embryonic mast cells has been described using direct immunological methods. Employing the previously characterized mast cell specific monoclonal antibodies AA4 and BGD6 [7], [8], [11], [12], MCcps (AA4−/BGD6+) were identified in the AGM region of rat embryos at E11.5. MCcps as well as MCps appeared in the liver at E12.5 and then went on to populate other peripheral sites.

The present study provides direct evidence that the AGM region is the site where embryonic MCcps originate. Previous indirect studies have suggested that the yolk sac constitutes the embryonic site of mast cell origin. Injection of yolk sac cells from mouse embryos at E9.5 days of gestation (corresponding to E11.5 in rat embryos) reconstituted the mast cells in the skin of W/W^v^ mice [15]. *In vitro*, yolk sac cells from E8.5 mouse embryos gave rise to mast cells progenitors [16]. On the other hand, our immunolocalization results revealed that at E8.5, the rat yolk sac does not contain AA4−/BGD6+ cells. MCcps were detected only at E11.5 and they were restricted to the AGM region. Therefore, in the previous studies, the mast cells obtained from the yolk sac were probably derived from pluripotent stem cells that are known to be present in the yolk sac [20].

The results from the present study demonstrate that the rat MCcps, first seen at E11.5 (E9.5 in mice), originate in the AGM during the initiation of definitive hematopoiesis in the embryo. These findings are in agreement with the current evidence that definitive hematopoiesis in human and mouse embryos begins in the paraaortic splanchnopleura (PSp)/AGM [17], [21]. The yolk sac and intraembryonic regions have both been shown to be sites of hematopoiesis. During embryonic development two distinct hematopoietic waves occur in the yolk sac [16], [22], [23] and it was originally thought that the yolk sac gave rise to the definitive hematopoietic cells in the embryo. In mouse embryos, prior to the establishment of fetal circulation, the yolk sac, but not the embryo itself shows a transient hematopoietic activity when placed in culture for short periods of time. Other studies also indicated that hematopoietic progenitors develop in the yolk sac prior to their appearance in the embryo [24], [25]. A second wave of hematopoiesis occurs in the yolk sac accompanying the onset of fetal circulation, and yolk sac derived erythroid and myeloid progenitors can be found shortly after in embryonic blood [16]. These studies suggested that the yolk sac is the source of the definitive hematopoietic cells in embryos. However, another line of evidence suggests that definitive hematopoiesis originates from an intraembryonic site and not in the yolk sac [20], [26]–[31]. The putative intraembryonic hematopoietic site was identified at the early somite-pair stages in mice as the PSp. As early as E8.5 the PSp/AGM was found to contain multipotent cells [32] and by the end of E10 the frequency of Colony Forming Units-Spleen in the AGM increased to levels comparable to those found in adult bone marrow [33]. By E10.5, the cells derived from the AGM are capable of long-term functional hematopoietic repopulation of adult recipients [34]. Furthermore, the first appearance of human embryonic hematopoietic stem cells is in the AGM region [35]. However, the exact contribution of the intra and extra embryonic sites of hematopoiesis has not yet been completely elucidated [36], [37].

The clusters of MCcps seen at E11.5 are similar to the intra-aortic clusters that are considered to be the morphological manifestation of hematopoietic stem cell (HSC) activity [21], [38]. These HSC clusters arise from the ventral surface of the dorsal aorta [39] and the maturation of the HSCs then occurs in large clusters [40]. The production of HSCs in the mouse embryo starts at E11. The generation of HSCs is extremely rapid and by the end of E11 all embryos contain definitive HSCs [39]. This HSC activity in the AGM is transient and ceases after E12 [41].

At E12.5 MCcps were found in the liver along with very immature orthochromatic mast cells expressing the gangliosides derivatived from GD1b (AA4+). This is the first time that the MCps were present in the embryos. The appearance of more mature AA4+ mast cells in the fetal liver suggests that AA4−/BGD6+ MCcps have migrated from the AGM region to the liver, where they differentiated and matured. This suggestion is supported by other studies that show that embryonic hematopoietic cells migrate from the AGM region, through the blood stream, to colonize the liver [34], [42].

Kitamura et al. [14] were not able to identify mast cells in the liver or other embryonic tissues of E13.5 and E14.5 mice presumably due to the lack of specific markers. Using mast cells specific markers, in the present study embryonic rat MCcps were identified as early as E11.5 in the AGM and as early as E12.5 in the liver. Through immunostaining, orthochromatic MCps (AA4+/BGD6+) could also be identified at E13.5 and E14.5 in various tissues. Metachromatic mast cells were found in rat embryos beginning at E15.5 in the caudal mesenchyme, in agreement with what has been previously reported [15], [43], [44]. Therefore without the use of specific markers, the origin, number and distribution of embryonic mast cells may be misinterpreted.

The embryonic MCcps described in this study are virtually identical to the MCcps found in adult rat and mouse bone marrow [7], [8]. The MCcps from adult bone marrow are AA4−, BGD6+, c-kit+, CD34+, and CD13+. The cells isolated from rat embryos at 11.5 days of gestation are BGD6+, c-kit+, CD34+, CD13+, AA4−, Thy-1−, CD40−, do not express FcεRI on their surface and do not bind IgE. In addition, RT-PCR results revealed that both the embryonic MCcps and the adult rat bone marrow MCcps express the alpha and beta subunits of the FcεRI and the rat mast cell proteases 1 and 5 mRNAs. Unexpectedly, significant levels of carboxypeptidase A mRNA are not expressed in the embryonic MCcp. Previously, a mast cell progenitor that lacked mouse mast cell carboxypeptidase A (mMC-CPA) was identified. In the presence of SCF plus IL-3 these cells became strongly immunoreactive for mMC-CPA, suggesting distinct sequential roles for SCF and IL-3 in mast cell development [45]. The MCcp isolated in the present study is still relatively undifferentiated and does not yet contain significant message for CPA.

Although the number of immunopurified MCcps was insufficient for mast cell reconstitution experiments, the number was sufficient for *in vitro* experiments in which these cells were cultured with SCF and IL3. After 21 days in culture, the isolated MCcps (AA4−/BDG6+), from E11.5 embryos, originated a cell population that contained 32.2%±3.5% metachromatic cells. More important, all of the cells expressed the α-subunit of FcεRI and bound IgE on the cell surface. The mast cell specific gangliosides were also expressed on the cell surface.

Taken together, the results of this study confirm the identity of the AA4−/BGD6+ cells in the AGM region of E11.5 embryos as MCcps. An understanding of the embryonic origin and development of mast cells, together with the characterization of their phenotype may lead to the development of new therapies for diseases in which alterations in the patterns of mast cell proliferation and differentiation occur, such as in mastocytomas and mastocytosis.

## Supporting Information

Table S1 **Characteristics of Rat AA4−/BGD6+ Mast Cells (MCcps) and AA4+/BGD6+ Mast Cells.** Mast cells were immunomagnetically isolated from E11.5 or adult rat bone marrow and either immunostained or subjected to PCR.
(DOCX)
