# Supplementary material for: Rat Embryonic Mast Cells Originate in the AGM
Source: PLoS One. 2013 Mar 7;8(3):e57862. doi: 10.1371/journal.pone.0057862 (PMC3591394; doi:10.1371/journal.pone.0057862)
Supplement: Table S1 — Characteristics of Rat AA4−/BGD6+ Mast Cells (MCcps) and AA4+/BGD6+ Mast Cells. Mast cells were immunomagnetically isolated from E11.5 or adult rat bone marrow and either immunostained or subjected to PCR. (DOCX) [file pone.0057862.s001.docx]

**Table S1:** Characteristics of Rat AA4-/BGD6+ Mast Cells (MCcps) and AA4+/BGD6+ Mast Cells

|  | **Rat Embryo E11.5** | **Bone Marrow Adult Rat** | **Bone Marrow Adult Rat** |
| --- | --- | --- | --- |
|  | *AA4-/BGD6+ MCcp* | *AA4-/BGD6+ MCcp* | *AA4+/BGD6+* |
| ***Antibody:*** | | | |
| mAb BGD6 | + | +**^(1)^** | +**^(1)^** |
| mAb AA4 | - | - | + |
| Anti-FcεRI | - | - | + |
| Anti-IgE | - | - | + |
| Anti c-kit | + | + | + |
| Anti-CD34 | + | + | + |
| ***mRNA:*** | | | |
| FcεRIα | + | +**^(2)^** | + |
| FcεRIβ | + | + | + |
| RMCP1 | + | + | + |
| RMCP5 | + | + | + |
| RCPA | ± | + | + |

Mast cells were immunomagnetically isolated from E11.5 or adult rat bone marrow and either immunostained or subjected to PCR.

1. Jamur M, Moreno A, Mello L, Souza Junior D, Campos M, et al. (2010) Mast cell

repopulation of the peritoneal cavity: contribution of mast cell progenitors versus

bone marrow derived committed mast cell precursors. BMC Immunology 11: 32.

2. Jamur MC, Grodzki ACG, Moreno AN, de Mello LFC, Pastor MVD, et al. (2001)

Identification and isolation of rat bone marrow derived mast cells using the mast

cell specific monoclonal antibody mAb AA4. J Histochem Cytochem 49: 219-228.
